# Assessing the predictability of fungicide resistance evolution through in vitro selection

**DOI:** 10.1007/s41348-024-00906-0

**Published:** 2024-04-12

**Authors:** Nichola J. Hawkins

**Affiliations:** NIAB, Cambridge, UK

**Keywords:** Fungicide resistance, Experimental evolution, Risk assessment, Resistance management

## Abstract

Plant pathogens are highly adaptable, and have evolved to overcome control measures including multiple classes of fungicides. More effective management requires a thorough understanding of the evolutionary drivers leading to resistance. Experimental evolution can be used to investigate evolutionary processes over a compressed timescale. For fungicide resistance, applications include predicting resistance ahead of its emergence in the field, testing potential outcomes under multiple different fungicide usage scenarios or comparing resistance management strategies. This review considers different experimental approaches to in vitro selection, and their suitability for addressing different questions relating to fungicide resistance. When aiming to predict the evolution of new variants, mutational supply is especially important. When assessing the relative fitness of different variants under fungicide selection, growth conditions such as temperature may affect the results as well as fungicide choice and dose. Other considerations include population size, transfer interval, competition between genotypes and pathogen reproductive mode. However, resistance evolution in field populations has proven to be less repeatable for some fungicide classes than others. Therefore, even with optimal experimental design, in some cases the most accurate prediction from experimental evolution may be that the exact evolutionary trajectory of resistance will be unpredictable.

## Fungicide resistance

Control of plant pathogenic fungi generally relies on a combination of genetically resistant plant varieties and chemical fungicides, in addition to cultural measures and in some cases biological control. However, pathogens have evolved to overcome multiple control measures, including the emergence of strains virulent on previously-resistant crop varieties, and resistance to fungicides. Plant pathogens have evolved resistance to at least eight different classes of fungicides, and resistance against the oldest group of single-site inhibitor fungicides, the MBCs, has been reported in over 90 different plant pathogenic fungi (Hawkins and Fraaije 2021). Therefore, more durable control of plant disease requires careful management of the risk of resistance.

Resistance management aims to slow the evolution of resistance, by reducing the selective pressure exerted by any one fungicide and by reducing the overall pathogen population and epidemic growth rate by other means. Resistance management strategies include consideration of fungicide choice and dose rate, the use of fungicides in mixtures or alternations, and integrated pest management approaches incorporating non-chemical control measures (Corkley et al. 2022).

The optimal combination of control measures depends on the risk level and the nature of resistance that would evolve against each fungicide. For example, a fungicide programme should use fewer treatments with higher-risk products; optimal dose rates will depend on the level of resistance; and mixing partners should not show cross-resistance. Furthermore, any resistance monitoring programmes could also be tailored to detect the expected levels of resistance across the main cross-resistance groups, and to detect specific mutations once known (Hawkins and Fraaije 2021).

However, resistance management is most effective when used before resistance has emerged in the field, and monitoring is most useful when it can provide early warnings before control failure. Therefore, in order to deploy the most effective resistance management and monitoring at the most effective time, we would need to anticipate the evolution of resistance before it occurs in field populations.

Experimental evolution can be used to predict fungicide resistance, but the accuracy of those predictions has varied. This review will consider the different methodological approaches used for experimental selection of resistance and their suitability for addressing different questions concerning the evolution of fungicide resistance in the field, and other factors that may influence the accuracy of evolutionary predictions.

## Experimental evolution

Many of the major fundamental questions in evolutionary biology were raised in relation to the history of life on Earth over geological time, which could not be replicated or observed within human lifespans. Experimental evolution, using small organisms with short lifespans under strong selection, has allowed evolutionary scenarios to be tested and replicated within far smaller spatio-temporal scales (Van den Bergh et al. 2018). Microbial systems can provide especially tractable models, with generation times of hours or days and a whole evolving population able to live within a culture flask. The best-known study of this type is the LTEE (Long-Term Evolution Experiment) in which multiple parallel *E. coli* lines are under selection for in vitro growth and nutrient utilisation, which has now been running for over 75,000 generations (Lenski 2023).

Unfortunately for agriculture and medicine, the evolution of resistance against pesticides and anti-microbials takes place far more rapidly than the evolutionary changes seen in the fossil record. For this reason, it is sometimes given as an example of “evolution in action” (Gillen et al. 2015; Powles and Yu 2010), in which evolution in general, and specific patterns such as parallel evolution, can be observed within a timescale of years rather than eons. However, experimental evolution can still prove useful, by allowing the selection of resistance to be studied at even greater speed (Palmer and Kishony 2013). The rapid evolution of resistance under experimental conditions can be used to predict resistance before it occurs in the field or clinic (Lloyd et al. 2020), or to test a range of different selective scenarios (Hegreness et al. 2008; Birnbaum et al. 2021), informing resistance risk assessments and management strategies.

Bacteria, or at least the fast-growing culturable species, had already been established as ideal models for general experimental evolution, and have also been used for antibiotic selection studies (Card et al. 2019; Palmer and Kishony 2013). For larger species with longer generation times, fewer generations and treatments can be tested. Some insecticide selection studies have been carried out with insect pests (Zoh et al. 2021, Idier, 2023) or with the non-target arthropod *Daphnia magna* (Jansen et al. 2015). For herbicide resistance, the microscopic alga *Chlamydomonas reinhardtii* has been used as a faster-cycling model (Reboud et al. 2007), in addition to selection experiments using the weed species themselves (Meyer et al. 2022). Many fungal pathogens, as with bacteria, can be grown in culture with relatively small size and fast generation times. Antifungal resistance has been experimentally evolved in the clinical pathogenic yeast *Candida albicans* (Cowen et al. 2000), with further studies in the model yeast *Saccharomyces cerevisiae* due to more genetic tools being available (Cowen et al. 2001). In filamentous fungi, general experimental evolution has been carried out with the model *Aspergillus nidulans* (Schoustra et al. 2006), and in the opportunistic human pathogen* Aspergillus fumigatus* (da Silva Ferreira et al. 2004). Experimental evolution of fungicide resistance has so far been conducted with the plant pathogens *Zymoseptoria tritici* and *Botrytis cinerea*, as discussed in more detail below.

## Applications of experimental evolution to predict fungicide resistance

The earliest in vitro selection of fungicide resistance was carried out as part of genetics studies, selecting resistant mutants to characterise fungicidal mode of action by identifying the target site (Borck and Braymer 1974), and to establish potential resistance mechanisms (Jung et al. 1992) after resistance had already been reported in plant pathogens in the field (Schroeder and Provvidenti 1969). Subsequent studies aimed to test whether similar mutations would be possible in other pathogens (Wheeler et al. 1995; Albertini et al. 1999), the start of more predictive uses of in vitro selection. Overall, mutagenesis and in vitro selection of MBC resistance produced a range of mutations in the target site-encoding gene β-tubulin, of which a smaller subset have proven important in the field (Hawkins and Fraaije 2016).

Now resistance risk assessments are required for new fungicides (EPPO 2015), and mutagenesis and selection studies are an established part of this, as seen for recently introduced SDHI (Scalliet et al. 2012) and QiI fungicides (Fouché et al. 2022). Total resistance risk is a product of the risk level of the fungicide, pathogen and agronomic management (Brent and Hollomon 2007), and therefore if mutagenesis studies indicate that a fungicide class has higher resistance risk, this must be mitigated by more stringent management guidelines, such as using only in mixtures and limiting the number of sprays per crop (EPPO 2015). Optimal mixtures also depend on the relative resistance risk of the component fungicides. Where one fungicide is at lower risk, maximising the level of disease control from the lower-risk fungicide and minimising the dose of the high-risk fungicide would give more durable control, whereas for two high-risk fungicides, a balanced mixture would achieve a better trade-off between selection by the two components (Hobbelen et al. 2013).

Establishing the exact resistance levels and cross-resistance patterns of potential resistance mutations can further inform optimal management strategies. For example, in most cases, lower doses result in slower selection of resistance against a given fungicide, but in some cases mutants with lower resistance factors can be controlled at a higher dose (Mikaberidze et al. 2017), and mixtures or alternations generally combine fungicides with different modes of action to avoid positive cross-resistance but it can be useful to also include multiple fungicides with the same mode of action if cross-resistance is incomplete (Jorgensen et al. 2018).

Experimental evolution can also be used to directly test different management strategies, such as mixtures (Ballu et al. 2021) or alternations (Ballu et al. 2023a) of different fungicides. In vitro selection provides a relatively rapid and compact way to test a wide range of management strategies, so it can be used to address questions where a shortage of experimental data has been identified, such as mixtures of high-risk fungicides (van den Bosch et al. 2014).

## Experimental considerations to replicate field evolution

**Fig. 1:**
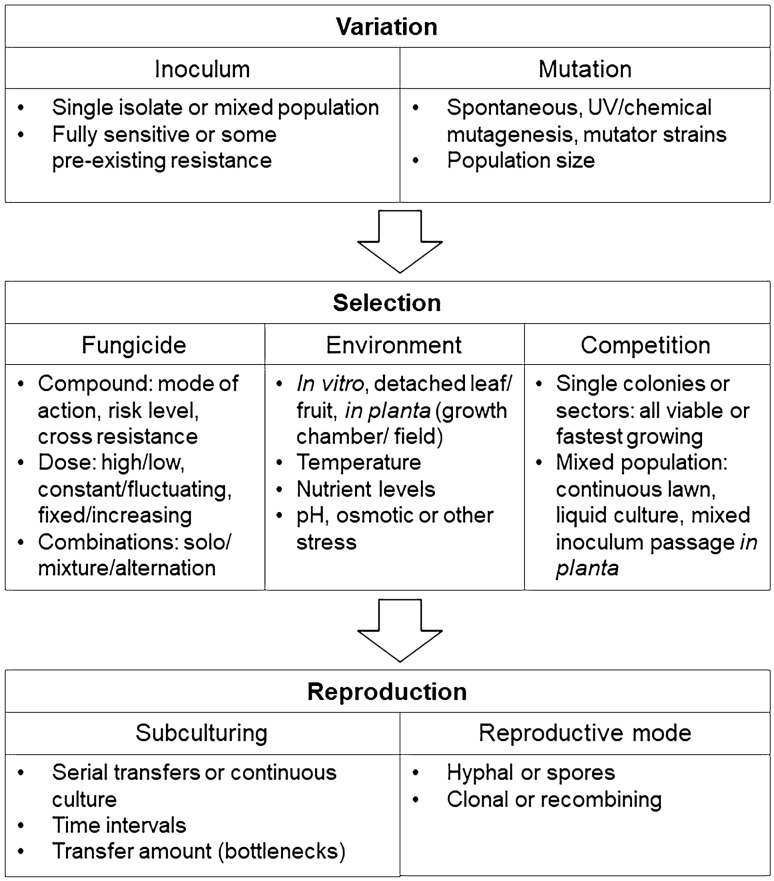
Factors to consider in experimental design for fungicide resistance selection

Evolution is the result of heritable variation under selection. Therefore, for experimental systems to replicate field evolution, both the available genetic variation and the selective pressure must be sufficiently similar. Some key methodological considerations are summarised in Fig. 1 and discussed below, and different experimental setups used in the broader field of microbial experimental evolution are described by Van den Bergh et al. (2018).

Each methodological choice has its own strengths and weaknesses in emulating different aspects of resistance evolution in the field, and ultimately, methodological choices should depend on the question being addressed. Are we trying to predict the exact mutations that will occur; or simply to assess whether the selection of any highly-resistant mutants is possible; or to use a model system for a general demonstration of the impacts of different management scenarios, regardless of whether the genetic basis of resistance is the same as in the field? Are we trying to anticipate potential new mutations that may be selected by a new mode of action, to manage ongoing selection of existing genotypes, or a combination of the two?

### Variation

Variation affecting fungicide sensitivity in field populations may be present within standing genetic variation, or it may arise by de novo mutations. A simple definition is that standing variants were present before the selection (in this case the fungicide) was introduced, whereas de novo mutations occurred afterwards, whether as a direct result of fungicide treatment or simply from ongoing spontaneous mutations (Hawkins et al. 2019). However, in practise, for a mutant to be seen as a standing variant it generally needs to already be present in multiple individuals that may have acquired further mutations or undergone recombination. Therefore, we can define a standing variant as already persisting through heredity in the population prior to selection, whereas a de novo mutation arises in a generation exposed to selection. This means de novo mutation does not necessarily require survival and mutation of sensitive genotypes post-treatment.

In field populations, fungicide target site mutations often arise de novo, but occasionally other resistance mechanisms such as copy number variants may be selected from standing variation (Hawkins et al. 2019; Steinhauer et al. 2019). Experimental evolution may start with a single parental isolate or a mixed inoculum. Starting with a single isolate means evolution depends entirely on mutations arising during the course of the experiment, so sufficient mutational supply is vital. Mutational supply can be increased with larger population sizes, with the obvious drawbacks that more space and media will be required, but experimental populations will always be orders of magnitude smaller than the total population of the pathogen in the field. Increasing the population size, in culture and during any transfer steps, can also reduce the risk of rare mutations being lost through genetic drift (Van den Bergh et al. 2018). Mutational supply can also be increased by UV or chemical mutagenesis, but this may result in different mutational biases from those found in field populations (Burns et al. 1986; Cano et al. 2023). However, for plant pathogens with highly genetically variable field populations (McDonald et al. 2022), experimental populations in which the mutational supply is too low for all possible resistance mutations to occur may also fail to reproduce the evolutionary pathways that occur in the field, or fail to give reproducible results between replicate lines so comparisons between treatments are not reliable. Gutiérrez-Alonso et al (2017) selected *Z. tritici* lines with SDHI fungicides, with and without UV mutagenesis. The UV-mutagenised lines underwent serial clonal replacement due to dose-dependent selection, indicating a trade-off between resistance levels and fitness penalties, whilst non-mutagenised lines had a single mutation or none. Therefore, the decision of whether to apply mutagenesis is a trade-off between the risk of unrepresentative mutational bias with mutagenesis, or unrepresentative mutational limitation without. Mutator strains could provide an alternative option without the same mutational biases as exogenous mutagenesis methods (Gifford et al. 2023).

Mutagenesis studies generally produce point mutations (Hawkins and Fraaije 2016), whereas other genetic mechanisms have been reported in the field including promoter inserts or copy number variants resulting in overexpression of the target site or efflux transporters (Lucas et al. 2015). In a small number of cases, fungicide resistant alleles appear to have been acquired through interspecific gene transfer (Chala et al. 2021; Hoffmeister et al. 2022), which can only be recreated in the laboratory under specific experimental designs (Morogovsky et al. 2022), but this is less common than for plasmid-borne antibiotic resistance and in most cases, recurring fungicide resistance across multiple species is the result of independent parallel evolution (Hawkins et al. 2019).

An alternative approach is to use a mixed inoculum, with a defined mix of known genotypes in set proportions (Chen et al. 2016; Fan et al. 2015). Using a defined mixed inoculum removes the stochasticity of mutation-limitation, which can make outcomes more repeatable with smaller populations. Variants can also be selected to high frequencies more rapidly from a defined fraction of the population, than from a new mutation that first arises in a single individual. This makes it well-suited to testing the impact of different selective scenarios on the dynamics of mutations that have already been reported in the field or predicted by mutagenesis experiments, but not for predicting the emergence of unknown new mutations. A defined mixed inoculum can also be used to emulate field populations, such as pre-existing resistance to single fungicides within a mixture (Ballu et al. 2023b).

### Selection

Usually in resistance evolution studies, the fungicide is intended to be the overriding selective pressure, but other conditions may also affect the outcome, especially if some resistant genotypes have specific fitness trade-offs such as temperature sensitivity.

Just as with fungicide use in the field, fungicide selection will depend on the choice of fungicide; dose rate and timing; and use of single fungicides, mixtures or alternations (Corkley et al. 2022). In risk assessment studies for a new fungicide, that fungicide must be used, but in other cases there is a choice. For testing more general evolutionary or management questions, fungicides may be chosen based on their resistance risk or the genetic basis of resistance, for example a high-risk fungicide with a target site encoded by a single nuclear gene; intensity of selection can be adjusted by using fungicides with different intrinsic activity as well as different doses; diversity of selection can be varied by mixing fungicides with and without cross-resistance; and heterogeneity of selection can be introduced by alternating different fungicides or treatment gaps.

Fungicide classes differ in their degree of cross resistance, which will determine whether one fungicide can be representative of the whole mode of action group or whether different compounds or subgroups would select different genotypes, but for a new mode of action, selection with multiple compounds may first be needed to establish this (Scalliet et al. 2012). In addition, different fungicides with the same mode of action may each select for non-cross-resistant genotypes when used alone, but combining them would select for alternative genotypes with positive cross-resistance. For some fungicides, different doses will also select different mutants (Gutiérrez-Alonso et al. 2017; Ballu et al. 2021), and the evolutionary dynamics of resistance may depend on whether selection experiments use a fixed dose throughout or increase the dose as populations adapt.

Some fungicide resistant genotypes carry fitness penalties, due to impaired function or altered stability of the target protein, or resource costs of overexpression or active transport (Hawkins and Fraaije 2018). Constant strong fungicide selection in vitro may outweigh any such fitness costs, whereas in field populations, genotypes with fitness penalties would be selected against during gaps in fungicide treatment, during or between growing seasons (Allen et al. 2017). This could be tested in vitro by selecting mutants in competing populations (Sect. "Competition"), and may be magnified with lower or fluctuating doses or treatment gaps, or environmental stressors such as temperature or lower nutrients. For example, some dicarboximide-resistant mutants have increased osmotic sensitivity (Beever 1983), and some β-tubulin mutants are heat or cold-sensitive (Thomas et al. 1985). However, fitness penalties relating to pathogenicity would only be shown *in planta*. For example, selection experiments have shown that in vitro, *Z. tritici* often loses accessory chromosomes, but this reduces fitness *in planta* (Möller et al. 2018). Experimental evolution *in planta* is possible, as shown in a study of host adaptation of plant pathogens on different cultivars and cultivar mixtures (Zhan and McDonald 2013), but at higher cost and lower speed and throughput than in vitro experiments.

Furthermore, detectable fitness costs are not always sufficient to prevent resistance emerging in the field. For example, temperature-sensitive β-tubulin mutations have been found in field isolates (Ma et al. 2003). This may reflect strong fungicide selection in that system, and further work is needed to establish what magnitude of fitness penalty could prevent or delay resistance under different levels of fungicide use and different durations of exposure versus gaps. Fitness costs could also be mitigated by compensatory mutations. Experimental evolution has been used to demonstrate possible mechanisms of fitness restoration in fludioxonil-resistant strains of *A. nidulans* (Dettman et al. 2017).

### Competition

Most in vitro selection studies of fungicide resistance so far have been simple mutagenesis experiments, with a single round of selection in which each mutant grew as a single-spore colony (e.g. Kendall et al. 1994; Albertini et al. 1999; Malandrakis et al. 2006; Ziogas et al. 2009; Scalliet et al. 2012) or a mutagenized colony was divided into individual mycelial plugs (Chen et al. 2012). This experimental design applies binary selection, and will select any mutants that are resistant to that fungicide dose and viable in those culture conditions. Conversely, “common garden” competition assays measure the overall selective advantage or disadvantage of genotypes in a defined starting population, quantifying the final frequencies by colony counts on selective plates or by molecular methods (e.g. Karaoglanidis et al. 2011; Chen et al. 2016). This experimental design applies quantitative selection, and mutants will increase in frequency if they have higher overall fitness in the presence of the fungicide.

However, the term “experimental evolution” is most widely used for experimental systems that include both mutation (whether mutagenized or spontaneous) and selection within mixed populations of competing mutants. This is usually from a single starting isolate per population, with mutants arising de novo during the experiment (e.g. Gutiérrez-Alonso et al. 2017; Fouché et al. 2022; Ballu et al. 2023a); but a mixed inoculum could also give rise to further mutants, if selection pressure strongly favours variants beyond those already present, such as tolnaftate-resistant mutants selected under combined azole and SDHI selection from a starting population containing only single-resistant, target-site mutants (Ballu et al. 2023b). Whilst in simple mutagenesis studies, mutants are grown on agar plates at a low enough density to form separate colonies, for mutants to compete they can either be grown on agar plates as a continuous lawn with transfer inoculum harvested from across the whole plate (Gutiérrez-Alonso et al. 2017), or in liquid culture (Fouché et al. 2022). At each subculturing step, an aliquot of the entire population is used as the inoculum for the following round of selection. Therefore, the proportion of each genotype will depend on its rate of growth or sporulation under those growth conditions in the presence of the fungicide. Mutants with higher fitness will increase in frequency in the population, and if multiple genotypes are insensitive to that fungicide dose, those with fitness penalties will be outcompeted by those without. An alternative approach to this “natural selection” is the “artificial selection” of faster-growing colonies or segments, but this proved less efficient for the selection of fludioxonil resistance in *A. nidulans* (Schoustra et al. 2005).

In addition, some studies have added “immigration”, with additional inoculum from outside of the evolving population added at transfer steps. For many pathogens in the field, gene flow is important in the spread of new genotypes, but experimental systems so far have primarily used the addition of further inoculum of the parental isolate to augment population numbers and prevent extinction in lines that have not yet adapted to the selective fungicide dose (Ballu et al. 2023a).

### Reproduction

In sexually reproducing pathogens or those with parasexual recombination, recombination can give rise to new combinations of resistance mutations, leading to the faster evolution of multiple resistance to different classes of fungicides (Taylor and Cunniffe 2022), or resistant alleles in fitter genetic backgrounds. Pathogens with a mixed reproductive mode have high adaptive potential, since they benefit from both repeated, high reproductive output from multiple clonal cycles during a growing season, and recombination during the sexual phase which may also produce airborne spores that increase gene flow (McDonald and Linde 2002).

In many species, the sexual cycle cannot be readily induced in vitro, so experimental evolution usually involves either reproduction via clonal conidia, or vegetative growth and propagation from hyphal tips. This replicates the clonal evolution seen in asexual pathogens, or within a growing season for pathogens with mixed reproductive mode. The lack of recombination can be compensated to some extent by increasing total mutational supply so mutations are more likely to recur in different backgrounds, or with mixed initial inoculum such that further mutations will occur in individuals that already have one mutation present.

For clonal experimental lineages, the choice of conidia or hyphal propagation can also affect evolutionary outcomes. In *A. fumigatus*, experimental evolution with serial transfers of asexual spores led to greater shifts in azole sensitivity than continuous hyphal growth in fungicide-amended agar over an equal time (Zhang et al. 2015). The authors suggest this may be due to a combination of higher mutational supply during the production of high numbers of conidia, and more effective selection of new mutations in haploid spores than multinucleate hyphae. Faster adaptation of haploid than diploid lines has been demonstrated using isogenic haploid and diploid strains of *A. nidulans* under fludioxonil selection (Schoustra et al. 2005), and diploid species also have lower resistance risk in the field for those fungicides where resistance is recessive (De Miccolis Angelini et al. 2015).

Fitness penalties can also vary between different fungal life stages or growth forms, due to physiological differences such as higher sterol requirements at some points in the fungal life cycle. For example, fenhexamid-resistant mutants of *B. cinerea* retained wild-type levels of mycelial growth, but had reduced conidial production and germination (Ziogas et al. 2003). In such cases, experimental evolution with hyphal or sporulating cultures could result in the selection of different mutations.

## Predicting predictability

The experimental factors described above can make in vitro predictions of fungicide resistance more representative of what is likely to happen in field populations. However, for some fungicide groups, field evolution itself is less inherently predictable, with a range of different target site mutations and other resistance mechanisms found within and between plant pathogen species (Hawkins and Fraaije 2021).

For the MBC fungicides, mutagenesis studies produced a range of β-tubulin mutations, of which a small subset have occurred most frequently in plant pathogens in the field (Hawkins and Fraaije 2016). In this case, the most useful improvement to predictions from experimental evolution would be to include competition and consider different selective conditions, to assess which mutations have higher overall fitness. For QoI fungicides, cytochrome *b* mutations conferring resistance have proven even more predictable in the field (Hawkins and Fraaije 2021), but less experimentally tractable as the target site is mitochondrially encoded.

However, for the azole and SDHI fungicides, resistance evolution in the field has been far less repeatable at the genotypic level (Hawkins and Fraaije 2021). In such cases, experimental evolution can still be useful, revealing some of the fitness trade-offs that contribute to the more variable evolutionary outcomes (Gutiérrez-Alonso et al. 2017), or whether different management strategies would select for different variants, such as alternations or mixtures favouring more generalist resistance than single fungicides (Ballu et al. 2023a).

Furthermore, where resistance evolves by the stepwise accumulation of multiples mutations, this additional complexity will reduce predictability, especially where there are epistatic interactions between the mutations so that later steps depend on the genotypes selected at earlier steps, combined with fitness trade-offs or incomplete cross-resistance affecting the selected mutations at each stage (Cools, Hawkins et al., 2013). In these cases, experimental evolution could predict the next step from a given starting genotype, but accurately predicting the full evolutionary pathway would require a detailed understanding of the selective conditions favouring not just each mutation individually but each combined genotype.

Further research will show whether evolutionary pathways that are more dependent on starting genotype or on specific selective conditions in the lab are also less predictable in the field, so that for future fungicide groups, risk assessments could assess not only what resistance mutations are possible, but whether or not we can reliably predict which individual mutations will be most significant in the field.

## References

[CR1] Albertini C, Gredt M, Leroux P (1999). Mutations of the beta-tubulin gene associated with different phenotypes of benzimidazole resistance in the cereal eyespot fungi *Tapesia yallundae* and *Tapesia acuformis*. Pestic Biochem Physiol.

[CR2] Allen RC, Engelstadter J, Bonhoeffer S, McDonald BA, Hall AR (2017). Reversing resistance: different routes and common themes across pathogens. Proc Royal Soc B-Biol Sci.

[CR3] Ballu A, Deredec A, Walker AS, Carpentier F (2021). Are efficient-dose mixtures a solution to reduce fungicide load and delay evolution of resistance?. Exp Evol Approach Microorg.

[CR4] Ballu A, Despréaux P, Duplaix C, Dérédec A, Carpentier F, Walker A-S (2023). Antifungal alternation can be beneficial for durability but at the cost of generalist resistance. Commun Biol.

[CR5] Ballu A, Ugazio C, Duplaix C, Noly A, Wullschleger J, Torriani SF, Walker AS (2022). Preventing multiple resistance above all: new insights for managing fungal adaptation. bioRxiv, 2022-12.. 10.1111/1462-2920.16614 38570900

[CR6] Beever RE (1983). Osmotic sensitivity of fungal variants resistant to dicarboximide fungicides. Trans Br Mycol Soc.

[CR7] Birnbaum SSL, Schulz NKE, Garrett DS, Tate AT (2021). Experimental evolution of insect resistance to two pesticide classes reveals mechanistic diversity and context-dependent fitness costs. *bioRxiv,* 2021.09.03.458899.

[CR8] Borck K, Braymer HD (1974). The genetic analysis of resistance to benomyl in *Neurospora*
*crassa*. J Gen Microbiol.

[CR9] Brent KJ, Hollomon DW (2007). Fungicide resistance: the assessment of risk. FRAC Monographs.

[CR10] Burns PA, Allen FL, Glickman BW (1986). DNA sequence analysis of mutagenicity and site specificity of ethyl methanesulfonate in Uvr+ and UvrB- strains of *Escherichia coli*. Genetics.

[CR11] Cano AV, Gitschlag BL, Rozhoňová H, Stoltzfus A, McCandlish DM, Payne JL (2023). Mutation bias and the predictability of evolution. Philos Trans Royal Soc B: Biol Sci.

[CR12] Card KJ, Labar T, Gomez JB, Lenski RE (2019). Historical contingency in the evolution of antibiotic resistance after decades of relaxed selection. *bioRxiv,* 695767. 10.1371/journal.pbio.3000397 PMC6827916 31644535

[CR14] Chen FP, Fan JR, Zhou T, Liu XL, Liu JL, Schnabel G (2012). Baseline sensitivity of *Monilinia fructicola* from China to the DMI fungicide SYP-Z048 and analysis of DMI-resistant mutants. Plant Dis.

[CR15] Chen SN, Luo CX, Hu MJ, Schnabel G (2016). Fitness and competitive ability of *Botrytis cinerea* isolates with resistance to multiple chemical classes of fungicides. Phytopathology.

[CR16] Cools HJ, Hawkins NJ, Fraaije BA (2013). Constraints on the evolution of azole resistance in plant pathogenic fungi. Plant Pathol.

[CR17] Corkley I, Fraaije B, Hawkins N (2022). Fungicide resistance management: maximising the effective life of plant protection products. Plant Pathol.

[CR18] Cowen LE, Kohn LM, Anderson JB (2001). Divergence in fitness and evolution of drug resistance in experimental populations of *Candida albicans*. J Bacteriol.

[CR19] Cowen LE, Sanglard D, Calabrese D, Sirjusingh C, Anderson JB, Kohn LM (2000). Evolution of drug resistance in experimental populations of *Candida albicans*. J Bacteriol.

[CR20] Da Silva Ferreira ME, Capellaro JL, Dos Reis Marques E, Malavazi I, Perlin D, Park S, Anderson JB, Colombo AL, Arthington-Skaggs BA, Goldman MH, Goldman GH (2004). In vitro evolution of itraconazole resistance in *Aspergillus fumigatus* involves multiple mechanisms of resistance. Antimicrob Agents Chemotherap.

[CR21] De Miccolis Angelini RM, Pollastro S, Faretra F, Ishii H, Hollomon DW (2015). Genetics of Fungicide Resistance. Fungicide Resistance in Plant Pathogens: Principles and a Guide to Practical Management.

[CR22] Dettman JR, Rodrigue N, Schoustra SE, Kassen R (2017). Genomics of compensatory adaptation in experimental populations of *Aspergillus*
*nidulans*. G3 Genes Genomes Genetics.

[CR23] EPPO (2015). PP 1/213 (4) resistance risk analysis. EPPO Bull.

[CR24] Fan Z, Yang JH, Fan F, Luo CX, Schnabel G (2015). Fitness and competitive ability of *Alternaria alternata* field isolates with resistance to SDHI, Qol, and MBC fungicides. Plant Dis.

[CR25] Fouché G, Michel T, Lalève A, Wang NX, Young DH, Meunier B, Debieu D, Fillinger S, Walker A-S (2022). Directed evolution predicts cytochrome *b* G37V target site modification as probable adaptive mechanism towards the QiI fungicide fenpicoxamid in *Zymoseptoria tritici*. Environ Microbiol.

[CR26] Gifford DR, Berríos-Caro E, Joerres C, Suñé M, Forsyth JH, Bhattacharyya A, Galla T, Knight CG (2023). Mutators can drive the evolution of multi-resistance to antibiotics. PLoS Genet.

[CR27] Gillen AL, Conrad J, Cargill M (2015). The genesis and emergence of community-associated methicillin-resistant *Staphylococcus aureus* (CA-MRSA): an Example of evolution in action?

[CR28] Gutiérrez-Alonso O, Hawkins NJ, Cools HJ, Shaw MW, Fraaije BA (2017). Dose-dependent selection drives lineage replacement during the experimental evolution of SDHI fungicide resistance in *Zymoseptoria tritici*. Evol Appl.

[CR29] Hawkins NJ, Fraaije BA (2016). Predicting resistance by mutagenesis: lessons from 45 years of MBC resistance. Front Microbiol.

[CR30] Hawkins NJ, Fraaije BA (2021). Contrasting levels of genetic predictability in the evolution of resistance to major classes of fungicides. Mol Ecol.

[CR31] Hawkins NJ, Bass C, Dixon A, Neve P (2019). The evolutionary origins of pesticide resistance. Biol Rev.

[CR32] Hawkins NJ, Fraaije BA (2018). Fitness Penalties in the Evolution of Fungicide Resistance. In: Leach JE & Lindow SE (eds.) *Annual Review of Phytopathology *Palo Alto: Annual Reviews 10.1146/annurev-phyto-080417-050012 29958074

[CR33] Hegreness M, Shoresh N, Damian D, Hartl D, Kishony R (2008). Accelerated evolution of resistance in multidrug environments. Proc Natl Acad Sci.

[CR34] Hobbelen PHF, Paveley ND, Oliver RP, Van Den Bosch F (2013). The usefulness of fungicide mixtures and alternation for delaying the selection for resistance in populations of *Mycosphaerella graminicola* on winter wheat: a modeling analysis. Phytopathology.

[CR35] Hoffmeister M, Mehl A, Hinson A, Siepe I, Taufferner T, Stammler G, Felsenstein F (2022). Acquired QoI resistance in *Pyrenophora teres* through an interspecific partial gene transfer by *Pyrenophora tritici-repentis*?. J Plant Dis Prot.

[CR36] Idier M, Siegwart M, Barrès B, Maugin S, Olivares J, Gauffre B (2023). Genetic characterization of multiple insecticide resistances in *Cydia*
*pomonella* (L.) using RNAseq. Entomologia Generalis.

[CR37] Jansen M, Coors A, Vanoverbeke J, Schepens M, De Voogt P, De Schamphelaere KAC, De Meester L (2015). Experimental evolution reveals high insecticide tolerance in *Daphnia* inhabiting farmland ponds. Evol Appl.

[CR38] Jorgensen LN, Matzen N, Hansen JG, Semaskiene R, Korbas M, Danielewicz J, Glazek M, Maumene C, Rodemann B, Weigand S, Hess M, Blake J, Clark B, Kildea S, Batailles C, Ban R, Havis N, Treikale O (2018). Four azoles’ profile in the control of Septoria, yellow rust and brown rust in wheat across Europe. Crop Prot.

[CR39] Jung MK, Wilder IB, Oakley BR (1992). Amino acid alterations in the benA (*β*-tubulin) gene of *Aspergillus nidulans* that confer benomyl resistance. Cell Motil Cytoskelet.

[CR40] Karaoglanidis GS, Luo Y, Michailides TJ (2011). Competitive ability and fitness of *Alternaria alternata* isolates resistant to QoI fungicides. Plant Dis.

[CR41] Kendall SJ, Hollomon DW, Ishii H, Heaney SP (1994). Characterisation of benzimidazole-resistant strains of *Rhynchosprium secalis*. Pestic Sci.

[CR42] Lenski RE (2023). Revisiting the design of the long-term evolution experiment with *Escherichia coli*. J Mol Evol.

[CR43] Lloyd DG, Schofield BJ, Goddard MR, Taylor EJ (2020). *De Novo* resistance to Arg10-teixobactin occurs slowly and is costly. Antimicrob Agents Chemotherap.

[CR44] Lucas JA, Hawkins NJ, Fraaije BA (2015). The evolution of fungicide resistance. Adv Appl Microbiol.

[CR45] Ma ZH, Yoshimura MA, Michailides TJ (2003). Identification and characterization of benzimidazole resistance in *Monilinia fructicola* from stone fruit orchards in California. Appl Environ Microbiol.

[CR46] Malandrakis AA, Markoglou AN, Nikou DC, Vontas JG, Ziogas BN (2006). Biological and molecular characterization of laboratory mutants of *Cercospora beticola* resistant to Qo inhibitors. Eur J Plant Pathol.

[CR47] McDonald BA, Linde CC (2002). Pathogen population genetics, evolutionary potential, and durable resistance. Annu Rev Phytopathol.

[CR48] McDonald BA, Suffert F, Bernasconi A, Mikaberidze A (2022). How large and diverse are field populations of fungal plant pathogens? The case of *Zymoseptoria tritici*. Evol Appl.

[CR49] Meyer L, Pernin F, Michel S, Bailly G, Chauvel B, Le Corre V, Délye C (2022). Lab meets field: accelerated selection and field monitoring concur that non-target-site-based resistance evolves first in the dicotyledonous, allergenic weed *Ambrosia*
*artemisiifolia*. Plant Sci.

[CR50] Mikaberidze A, Paveley N, Bonhoeffer S, Van Den Bosch F (2017). Emergence of resistance to fungicides: the role of fungicide dose. Phytopathology.

[CR51] Möller M, Habig M, Freitag M, Stukenbrock EH (2018). Extraordinary genome instability and widespread chromosome rearrangements during vegetative growth. Genetics.

[CR52] Morogovsky A, Handelman M, Abou Kandil A, Shadkchan Y, Osherov N (2022). Horizontal gene transfer of triazole resistance in *Aspergillus fumigatus*. Microbiol Spectr.

[CR53] Palmer AC, Kishony R (2013). Understanding, predicting and manipulating the genotypic evolution of antibiotic resistance. Nat Rev Genet.

[CR54] Powles SB, Yu Q (2010). Evolution in action: plants resistant to herbicides. Annu Rev Plant Biol.

[CR55] Reboud X, Majerus N, Gasquez J, Powles S (2007). *Chlamydomonas reinhardtii* as a model system for pro-active herbicide resistance evolution research. Biol J Lin Soc.

[CR56] Scalliet G, Bowler J, Luksch T, Kirchhofer-Allan L, Steinhauer D, Ward K, Niklaus M, Verras A, Csukai M, Daina A, Fonné-Pfister R (2012). Mutagenesis and functional studies with succinate dehydrogenase inhibitors in the wheat pathogen *Mycosphaerella graminicola*. PLoS ONE.

[CR57] Schoustra SE, Slakhorst M, Debets AJM, Hoekstra RF (2005). Comparing artificial and natural selection in rate of adaptation to genetic stress in *Aspergillus nidulans*. J Evol Biol.

[CR58] Schoustra SE, Debets AJM, Slakhorst M, Hoekstra RF (2006). Reducing the cost of resistance; experimental evolution in the filamentous fungus *Aspergillus nidulans*. J Evol Biol.

[CR59] Schroeder WT, Provvidenti R (1969). Resistance to Benomyl in powdery mildew of cucurbits. Plant Disease Reporter.

[CR60] Steinhauer D, Salat M, Frey R, Mosbach A, Luksch T, Balmer D, Hansen R, Widdison S, Logan G, Dietrich RA, Kema GH, Bieri S, Sierotzki H, Torriani SF, Scalliet G (2019). 2019 A dispensable paralog of succinate dehydrogenase subunit C mediates standing resistance towards a subclass of SDHI fungicides in *Zymoseptoria*
*tritici*. PLoS Pathog.

[CR61] Taylor NP, Cunniffe NJ (2022). Optimal resistance management for mixtures of high-risk fungicides: robustness to the initial frequency of resistance and pathogen sexual reproduction. Phytopathology.

[CR62] Thomas JH, Neff NF, Botstein D (1985). Isolation and characterization of mutations in the beta-tubulin gene of *Saccharomyces cerevisiae*. Genetics.

[CR13] Turo C, Mair W, Martin A, Ellwood S, Oliver R, Lopez-Ruiz F (2021) Species hybridisation and clonal expansion as a new fungicide resistance evolutionary mechanism in *Pyrenophora teres *spp. bioRxiv

[CR63] Van den Bergh B, Swings T, Fauvart M, Michiels J (2018). Experimental design, population dynamics, and diversity in microbial experimental evolution. Microbiol Mol Biol Rev.

[CR64] Van Den Bosch F, Paveley N, Van Den Berg F, Hobbelen P, Oliver R (2014). Mixtures as a fungicide resistance management tactic. Phytopathology.

[CR65] Wheeler IE, Kendall SJ, Grosjean-Cournoyer MC, Butters JA, Hollomon DW, Dixon GK, Copping LG, Holloman D (1995). Molecular analysis of benzimidazole resistance in *Rhynchosporium secalis*. Antifungal Agents: Discovery and Mode of Action.

[CR66] Zhan J, McDonald BA (2013). Field-based experimental evolution of three cereal pathogens using a mark- release-recapture strategy. Plant Pathol.

[CR67] Zhang J, Debets AJM, Verweij PE, Melchers WJG, Zwaan BJ, Schoustra SE (2015). Asexual sporulation facilitates adaptation: the emergence of azole resistance in *Aspergillus fumigatus*. Evolution.

[CR68] Ziogas BN, Markoglou AN, Malandrakis AA (2003). Studies on the inherent resistance risk to fenhexamid in *Botrytis cinerea*. Eur J Plant Pathol.

[CR69] Ziogas BN, Nikou D, Markoglou AN, Malandrakis AA, Vontas J (2009). Identification of a novel point mutation in the beta-tubulin gene of *Botrytis cinerea* and detection of benzimidazole resistance by a diagnostic PCR-RFLP assay. Eur J Plant Pathol.

[CR70] Zoh MG, Bonneville J-M, Tutagata J, Laporte F, Fodjo BK, Mouhamadou CS, Sadia CG, McBeath J, Schmitt F, Horstmann S, Reynaud S, David J-P (2021). Experimental evolution supports the potential of neonicotinoid-pyrethroid combination for managing insecticide resistance in malaria vectors. Sci Rep.

